# Effects of exercise on the sleep microarchitecture in the aging brain: A study on a sedentary sample

**DOI:** 10.3389/fnsys.2022.855107

**Published:** 2022-10-26

**Authors:** Tuan Z. Cassim, Keith M. McGregor, Joe R. Nocera, Violet V. García, Christopher G. Sinon, Matthias Kreuzer, Paul S. García

**Affiliations:** ^1^Department of Anesthesiology, Neuroanesthesia Division, Columbia University Medical Center, New York-Presbyterian Hospital, New York, NY, United States; ^2^Department of Clinical and Diagnostic Sciences, School of Health Profession, University of Alabama at Birmingham, Birmingham, AL, United States; ^3^Birmingham Veterans Affairs Geriatric Research, Education, and Clinical Center (GRECC), Birmingham, AL, United States; ^4^VA Rehabilitation R&D Center for Visual and Neurocognitive Rehabilitation, Atlanta VAMC, Decatur, GA, United States; ^5^Department of Neurology, Emory University School of Medicine, Atlanta, GA, United States; ^6^Department of Rehabilitation Medicine, Emory University School of Medicine, Atlanta, GA, United States; ^7^Stamps President’s Scholars Program, Georgia Institute of Technology, Atlanta, GA, United States; ^8^Yerkes National Primate Research Center, Neuropharmacology and Neurologic Diseases, Emory University, Atlanta, GA, United States; ^9^Department of Anesthesiology, School of Medicine, Technical University of Munich, Munich, Germany

**Keywords:** aging, aerobic exercise, physical activity, sleep, deep sleep (slow wave sleep, SWS), electroencephaloagraphy (EEG)

## Abstract

Having a healthy sleep pattern plays a vital role in one’s overall health. Sleep in the elderly is characterized by decreased slow-wave sleep and an increase of REM sleep. Furthermore, quantitative electroencephalographic (qEEG) studies have shown an age-related attenuation of total EEG power in sleep. However, exercise has been shown to improve sleep across all age groups. In this study, we used the Sleep Profiler™ EEG Sleep Monitor to observe EEG changes occurring during sleep following an aerobic exercise intervention. This study was done on older adults (*N* = 18, with only five subjects containing both pre- and post-data of sufficient quality for analysis) with an age range 60–85 years. The aerobics regimen was performed three times weekly for 12-weeks commencing with 20-min sessions. The time of each session progressed by 1–2 min/session as needed to a maximum time of 45 min per session. The macro-architecture (sleep stages) and microarchitecture (EEG) results were analyzed using MATLAB. For the microarchitecture, our results showed more deep sleep following the aerobic exercise regimen. Furthermore, for the microarchitecture, out results shows an increase in total EEG power post-exercise in both light (N1 and L1) and deep sleep (N2 and N3). These preliminary changes in sleep the microarchitecture suggest that non-pharmacologic methods might mitigate age-related EEG changes with potential implications for neurocognitive health.

## Introduction

Sleep, regulated by circadian and homoeostatic processes plays a vital role in one’s overall health (Borbely et al., 2016). The sleep architecture in humans consists of intermittent cycling among four sleep “stages” which are distinguished from one another *via* neurophysiological recording of muscle and brain activity. The ratios of these stages on a given night of sleep for an individual vary based on many factors such as age, sex, ethnicity, smoking habits, alcohol consumption, etc. (Sahlin et al., 2009). Studies categorize sleep architecture into vigilance stages also known as the macro-architecture and spectral analysis of the electroencephalographic (EEG) also known as the micro-architecture to understand variables associated with sleep. Ocular movements distinguish rapid eye movement (REM) EREM (rapid eye movement) sleep from the three types of non-REM sleep: non-REM stage 1, N1; non-REM stage 2, N2; non-REM stage 3, N3. Unique EEG features pertaining to these stages and their analyses are well documented in sleep literature (Silber et al., 2007). High frequency, low amplitude neurophysiological activity in scalp electrodes over the frontal cortex are indicative of N1 and REM—so called “light” stages of sleep, because arousal is common with mild stimuli (e.g., auditory stimulation at normal volume). Stimuli of higher intensity (e.g., loud voice, tactile stimulation) are necessary to arouse an individual from N2 to N3 sleep, which are characterized by higher amplitude lower frequency synchronized oscillations over frontal electrodes (Schulz, 2008). Neurophysiological recordings serve as reliable biomarkers of sleep stages even when recorded *via* portable at-home sleep devices (Levendowski et al., 2017).

With aging, sleep processes can be disrupted causing unhealthy sleep patterns and sometimes influence pathogenesis of sleep disorders (Zhong et al., 2019). In older adults, complaints about sleep include: light sleep, spontaneous waking during the night, daytime sleepiness, and a decrease in overall sleep time (Bliwise et al., 1992; Foley et al., 1995; Vitiello et al., 2004). Sleep disorders are also associated with cognitive decline (Dzierzewski et al., 2018), yet they remain a commonly underdiagnosed problem. In the literature, slow wave sleep (SWS) also known as deep sleep has shown to be a crucial biomarker that is associated with restorative properties such as memory consolidation and improvements in age-related memory integrity (Papalambros et al., 2017; Berres and Erdfelder, 2021). However, one of the most prevalent sleep problems observed in the aged population is an increase in light sleep (N1 and N2) and a decrease in SWS (Scullin, 2013; Scullin and Bliwise, 2015). Quantitative EEG (qEEG) studies have furthered the understanding of age-related changes in sleep by decoding specific spectral feature changes in the sleep EEG (Carrier et al., 2001). Non-pharmacological methods to improve SWS in this population is of special interest and can have positive implication in the clinical setting and in one’s overall health.

Exercise has shown to have beneficial effects to mitigate age-related health problems. Exercise has also shown to have neuroprotective qualities and help the cognitively vulnerable populations and attenuate cognitive problems (Heyn et al., 2004; Voss et al., 2013). The beneficial effects of exercise on sleep are also well documented in the literature (Kredlow et al., 2015). Aerobic activity in particular has shown to have promising results in promoting healthy sleep patterns and playing a positive role in brain health (Hillman et al., 2008; Kaliman et al., 2011; Hotting and Roder, 2013). Animals studies have also shown positive effects of aerobic exercise has on sleep in aged mice (Panagiotou et al., 2018). Thus, as an alternate to pharmacological interventions for sleep disturbances exercise has been proposed as a non-pharmacological intervention tool to mitigate sleep problems (Chennaoui et al., 2015).

Lastly, advanced age also affects the inhibitory signaling of cortical networks (Lariviere et al., 2007). The efficacy of these inhibitory cortical networks strongly correlates with sleep quality (Paul-Savoie et al., 2012). Exercise can reverse the loss of inhibitory signaling among cortical networks to improve neurologic performance (McGregor et al., 2017). Hence, an increased level of physical fitness can lead to improved sleep quality (King et al., 2008) in older adults that were previously sedentary.

While there are numerous reports of the human and animal studies of potential neuroprotective effect of acute exercise through improved sleep (Kredlow et al., 2015; Panagiotou et al., 2018), the detailed changes of aerobic exercise have on sleep microarchitecture (i.e., quantitative EEG features) is not well studied. Further, we know of no studies that have investigated quantitative EEG in older adults after a longer-term physical activity intervention. In our study, we demonstrate the feasibility of an “in-home” device for capturing neurophysiologic data during natural sleep in older adults while we also examined the effects of aerobic exercise on the sleep microarchitecture we decode changes in the spectral features in this aged sample. Given the evidence from the literature, we expect to see an improvement in SWS associated with aerobic exercise.

## Methods

### Experimental design

Approval for this study was granted by the local Institutional Review Board and written informed consent was obtained from each participant in the study in accordance with the Declaration of Helsinki. This study was a sub-study of a larger study (McGregor et al., 2017; Nocera et al., 2017) comparing the effects of 12-weeks of aerobic exercise (bicycle “spin” class, three times each week) vs. 12-weeks of non-aerobic stretching and balance “control” condition on motor responsiveness. A total of 28 separate participants between the ages of 60–89 took a Sleep Profiler™ EEG sleep monitor (a battery-powered, wireless, and home-health sleep profiling device) home at least once for collecting sleep data. Not all participants were able to reliably provide data for the study. For our analysis we included a total of *n* = 18 with baseline (pre-aerobic intervention) EEGs. To investigate the effect of exercise on sleep we had *n* = 5 EEG complete sets of both pre- and post-aerobic exercise recordings of sufficient quality for analysis of microarchitecture. Due to acquisition limitations on the device, stable EEG data was collected from 18 out of 28 participants and of these 18 only 5 participants were able to provide pre/post intervention data.

### Subjects

Our participants (aged 60–85 years) were sedentary [defined as engaging in less than 45 min of weekly moderate to vigorous physical activity as assessed by the International Physical Activity Questionnaire (IPAQ)], without major psychiatric or neurological disease, -right-handed, and native English speakers. Participants also required physician’s approval for study participation. No participants had been hospitalized within the previous 6 months or exhibited significant cognitive impairment defined as < 23 on the Montreal Cognitive Assessment (MoCA) (Nasreddine et al., 2005) and < 15 errors on the American National Adult Reading Test (Grober and Sliwinski, 1991).

### Exercise regimen

Consistent with our previous work (Nocera et al., 2017; McGregor et al., 2018), the spin and control groups engaged in exercise interventions beginning with 20 min of activity three times a week for 12 weeks and led by a qualified instructor. In both groups, the time of each session progressed based on the recommendation of the instructor by 1–2 min to a maximum time of 45 min per session at the end of the 12 weeks. For all participants, we calculated their estimated maximum heart rate using the Karvonen method (Eq. 1), and then calculated heart rate reserve (HRR) as shown in Eq. 2. We used HRR values to evaluate physical workload of participants throughout the 12-week interventions.


(1) Max⁢Heart⁢Rate=220⁢bpm-Age

(2) Heart⁢Rate⁢Reserve=Max⁢Heart⁢Rate-Resting⁢Heart⁢Rate

For the spin group, exercise intensity began at low levels (target: 50% of HRR) and increased by 5% every week (as deemed appropriate by the instructor) to a target maximum of 80–85% HRR. Participants wishing to exceed this capacity could do so for limited exercise intervals. Relative physical exertion was assessed with the “talk-test” and a rating of relative physical exertion estimation using the Borg 6–20 difficulty scale (6 = lowest effort; 20 = maximum effort) during the sessions.

For the spin group the instructor guided the participants through a light 5-min warm up, then a workout phase that included steady up-tempo cadences, sprints (increased rpm), and climbs (increased resistance). As such, the spin exercise routine employed an interval-based training approach. During the workout phase, the target HRR was maintained by averaging increases and decreases in intensity/HR. The goal was to maintain within a 10% offset from the HRR goal during the workout phase. For the control group, we monitored heart rate, but did not specify a target HRR threshold for the balance (progressive board/mat balance), stretching and light strength training (using resistance bands) exercises employed.

All interventions took place in a climate-controlled fitness facility. Interventions occurred in the mornings (8–10 a.m. Eastern Time Zone) and ran on a rolling basis throughout the calendar year.

### Sleep profiler and electroencephalographic recording

We used the Sleep Profiler™ EEG Sleep Monitor (Advanced Brain Monitoring, Carlsbad, CA, United States), a battery-powered, wireless, and home-health sleep profiling device. Technical details regarding the device, sampling rates, processing, and filtering can be accessed in the company website.^1^ This device has been validated for staging of sleep and is FDA cleared for aiding in the diagnosis of sleep disorders (Levendowski et al., 2017). Participants took the device home after being instructed on its use. Participants were asked to clean their forehead using soap and water prior to the placement of the electrodes to minimize impedance. If the impedance level was too high a voice message alerted the participants to adjust the sensors. The frontal EEG data were recorded as a voltage difference between the two frontopolar electrode positions AF7–AF8, according to the international 10–20 system (sampling rate = 256 Hz). The system also recorded electrooculographic and electromyographic activity. The record of each subject was uploaded onto the Sleep Profiler Portal™, an internet-based software application. Each record was processed using an automatic scoring algorithm consistent with published guidelines American Academy of Sleep Medicine. Consistent with previous literature (Levendowski et al., 2017), adjustments for age-appropriate ratios of sleep stages were made. As is common in evaluating patients for sleep apnea, the sleep profiler sub-categorizes N2 into two separate stages (L2 and N2). Sleep epochs were scored as L2 if markers indicative of a lighter stage of sleep (e.g., elevated EMG activity or brief cortical arousals) were also present during N2 (Levendowski et al., 2017). The EEG recordings were stored in the edf format for further analysis in MATLAB (MathWorks, Natick, MA, United States).

### Sleep macro-architecture

Based on the device-derived sleep stages from Sleep Profiler™, we assessed the sleep macro-architecture (i.e., sleep stages) during the baseline recordings in our 18 participants. For the five participants included in the exercise group with valid pre- and post-intervention recordings, we also calculated the change in the recording time percentage, i.e., the pre/post change in percentage of sleep stages. Because we were especially interested in the amount of restorative sleep (deep sleep stages) rather than ratios of each individual stage of sleep, we combined the *light sleep* stages (N1 and L2) and *deep sleep stages* (N2 and N3).

### Sleep stages

To investigate a possible impact of exercise on the spectral information of the EEG, we calculated the power spectral density (PSD) and the density spectral arrays (DSA) with the MATLAB *pwelch* function with a frequency resolution of 1 Hz for the 30 s EEG segments the auto-profiler used to generate the sleep scores. In order to minimize the impact of segments that may contain a transition from one state to the other, we dismissed these epochs from quantitative EEG analysis.

### Statistical analysis

We performed all statistical analyses using MATLAB. We held the significance threshold at alpha = 0.05.

#### Sleep stages

Due to a limited sample size we chose the area under the receiver operating curve (AUC) with 10k-fold bootstrapped 95% confidence intervals (CI) of the relative change in the parameter from pre- to post-conditions as a measure of effect size. If the 95% CI excluded 0.5, we considered the effect statistically significant and if the 95% CI included 0.5, but the AUC was higher 0.7, we considered the effect to be clinically relevant (Mandrekar, 2010). Furthermore, to evaluate the distribution of the vigilance states, we calculated the Friedman test with *post-hoc* Tukey-Kramer correction. It has to be noted that for the analyses of the matched pre-pot data the results from the statistical analysis only carry little information because of the small sample size.

#### Spectral electroencephalographic features

In order to evaluate significant or relevant changes from the PSD analyses which are presented in Figures 3, 4, we only considered differences that occur in at least two neighboring frequencies (2 Hz window), an approach that was used earlier (Kreuzer et al., 2020). For AUC calculation with 10k-fold bootstrapped 95% confidence intervals, we used the MATLAB-based MES toolbox. We used this approach to evaluate possible differences within groups between pre- and post-exercise conditions as well as to find differences in the EEG recorded post-exercise between the two intervention groups.

## Results

### Sleep macro-architecture

**FIGURE 1:**
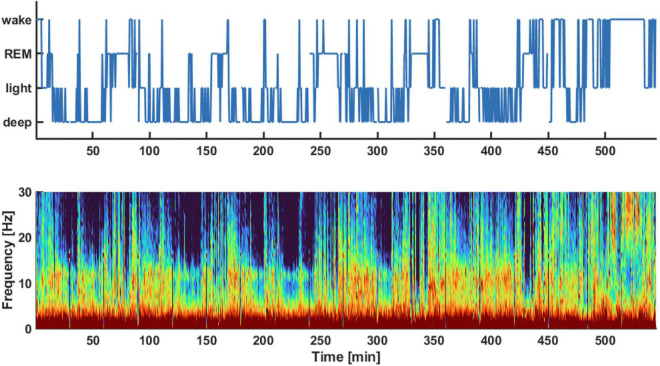
Exemplary hypnogram **(Top)** and density spectral array **(Bottom)** of the pre-aerobics intervention EEG recording session. The hypnogram on top shows the different vigilance states the subject transitioned throughout the night. These sleep stages were derived from the spectral information provided in the density spectral array. The auto staging details of the sleep profiler are described in Levendowski et al. (2017). For example, light sleep was indicated if there was strong activity in the EEG alpha-band around 10 Hz as indicated by red colors in this frequency range.

Figure 1 displays an exemplary hypnogram (1A) and corresponding spectral array (1B) for a typical participant in the sample. The corresponding hypnogram including the classical sleep stages is presented as Supplementary Figure 1. The frontal EEG data from the first to last sleep score for all (*n* = 18) participants included in the study yielded approximately 635 (480, 898) min [median (1st, 3rd quartile)] of sleep for each of the participants.

**FIGURE 2:**
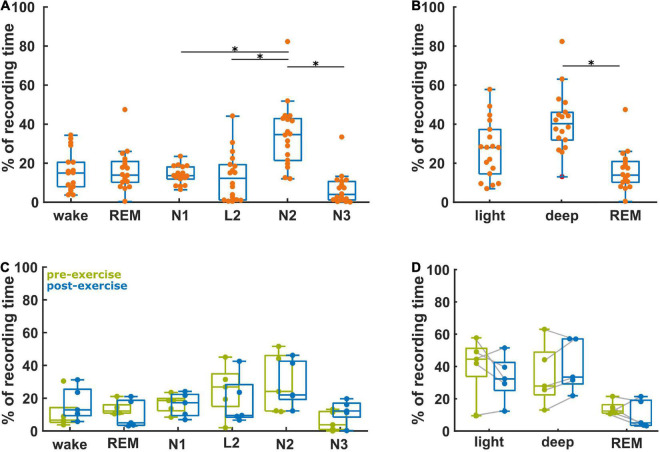
Proportion of the vigilance states derived from the baseline recordings (*n* = 18) and the ratio of the vigilance states between the baseline and post-aerobic exercise recordings from five participants. **(A)** Distribution of vigilance states for all 18 baseline recordings. **(B)** Distribution of *light sleep* (N1 + L2), *deep sleep* (N2 + N3) and *REM sleep* at baseline (*n* = 18). **(C)** Relative change of the vigilance states from baseline to post-aerobic exercise recordings (*n* = 5). **(D)** Relative change of *light sleep, deep sleep* and *REM sleep* from baseline to post-aerobic exercise recordings (*n* = 5). *Represent the TCassim.

Figure 2A presents the relative fraction of the different sleep stages for these 18 participants. The distribution of stages was significantly different (*p* < 0.001), with significantly higher proportions of N2, when compared to N1 (corrected *p* = 0.038), to N3 (*p* < 0.001), and to L2 (*p* = 0.001). The participants spent 28% (15, 37%) in light sleep, 14% (10, 21%) in REM sleep, and 40% (32, 46%) in deep sleep (Figure 2B). This distribution was significantly different (*p* < 0.001) with significantly more deep sleep than REM (*p* < 0.001). Figure 2C compares the vigilance state recording time percentage between baseline and after aerobic exercise for the five participants in the subgroup analysis. We did not find any significant differences in the distribution of the data, but four out of these five participants spent more time in deep sleep after the intervention [AUC = 0.8 (0.4, 1)]. There was no difference in light sleep [AUC = 0.6 (0.2, 1)] (Figure 2D). Further, four out of five participants spent less time in REM sleep after the intervention [AUC = 0.8 (0.4, 1)], Figure 2D. It appears that exercise resulted in more deep sleep (and less REM) in this small subgroup with pre- and post- intervention EEG data. Supplementary Table 1 contains the results from the statistical tests.

### Sleep micro-architecture

**FIGURE 3:**
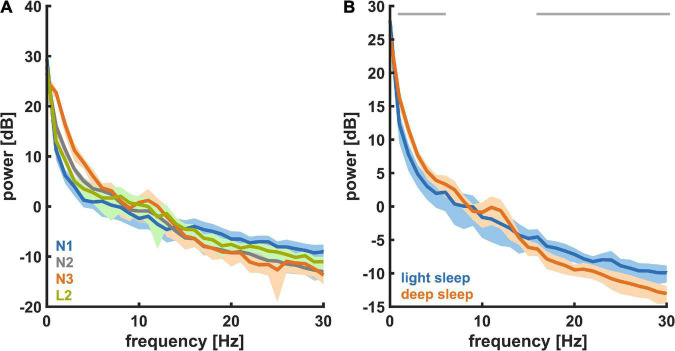
Power spectral density (PSD) for the different NREM sleep states recorded at baseline (*n* = 18). **(A)** For different levels of NREM sleep, i.e., N1, L2, N2, and N3. **(B)** For *light sleep* and *deep sleep*. During *deep sleep* the power in the low (delta) frequencies was significantly higher and the power in the high (beta) frequencies was significantly lower.

**FIGURE 4:**
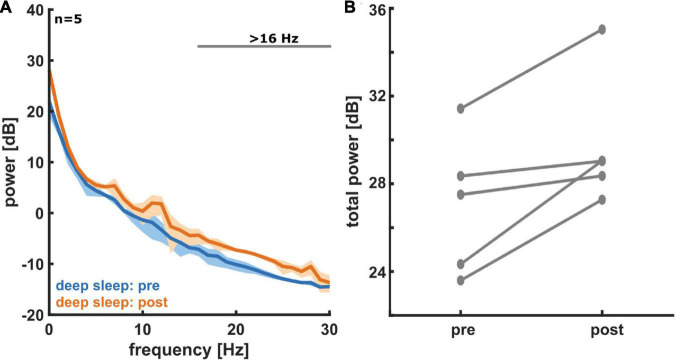
**(A)** Power spectral density (PSD) plots for deep sleep, both pre- (blue) and post- (orange) aerobic intervention (*n* = 5). A histogram for the measured EEG power in each frequency bin is displayed. Solid lines represent average for all five participants and shading represents 95% confidence interval. In deep sleep we see an increase in EEG power post-exercise, especially at moderate to high frequencies. **(B)** A comparison of total non-REM EEG power pre- and post-aerobic intervention (*n* = 5). For non-REM sleep states (light and deep sleep), we see an increase in total EEG power from pre- to post-aerobic intervention in frontal electrodes. Each pair of connected dots represents a single participant.

Figure 3A shows the PSD plots representing the power in each EEG frequency for the different NREM sleep stages and Figure 3B presents the PSD for *light sleep* and *deep sleep*. As expected, for these 18 participants (pre-aerobic intervention) the lighter stages of sleep (N1 + L2) have a more uniform distribution of EEG frequency, characterized by lower amplitude at the lower frequencies (< 5 Hz) and higher amplitude at the higher frequencies (> 14 Hz) as compared to deep sleep (N2 + N3). The horizontal gray lines at the top of the figure depict differences in EEG frequency that are determined to be statistically significant (see Methods). In Figure 4 we compare the spectral EEG features of *deep sleep* before and after the aerobic intervention in the five participants in our intervention group. Changes in the moderate and high frequencies predominate for *deep sleep* as depicted in Figure 4A. For the five participants with the matched EEG data, we observed a total EEG power increase during in all five participants for non-REM sleep (Figure 4B).

## Discussion

Our study confirmed results from previous literature that showed an association between physical activity and an improvement in sleep quality in older subjects. Specifically, our aerobic exercise intervention resulted in increased restorative sleep and an associated decrease of REM sleep in our participants. This novel finding regarding vigilance states, however, should still be considered somewhat preliminary given our modest sample size. Additionally, the change in sleep macro-architecture corresponded with changes in sleep microarchitecture. Namely, the exercise intervention led to an increase in overall EEG power in non-REM sleep. This is the first time an FDA-approved take-home sleep device has been used to examine the details of sleep neurophysiology in aerobic exercise interventions in a vulnerable, aged population.

Our findings are preliminary but provide some insight into the complex relationship between aging, exercise, sleep, and cognition. Total EEG power while sleeping, is known to be lower in older individuals (Carrier et al., 2001). Similarly, aged individuals and those with neurocognitive impairment also exhibit a decline in overall EEG power (Kreuzer et al., 2020). This might be related to an increased susceptibility to disturbances of attention and arousal, especially in clinical situations where anesthesia or sedation is used (e.g., post-surgery). Our results confirm EEG findings from other human studies (Petit et al., 2004) and indicate that aerobic exercise might restore beneficial sleep EEG patterns. It is possible that these EEG changes could indicate a potential therapeutic benefit of aerobic exercise on cognition and a potential non-pharmacologic approach could mitigate age-related attenuation of frontal EEG power during sleep.

Other researchers have demonstrated that 1 h of vigorous exercise can increase slow-wave sleep and decrease wake transitions in a small study consisting of young, healthy, and male subjects (Park et al., 2021). Our results are particularly note worthy as they show benefits for restorative sleep in older sedentary individuals with exercise. Six months of exercise was shown to increase sleep efficiency and modulate immune biomarkers in previously sedentary elderly subjects (Abd El-Kader and Al-Jiffri, 2019). Our 12-week regimen demonstrated benefit in a similar population. While none of our participants had a diagnosis of a pre-existing sleep disorder, these are often under diagnosed and more common as we age. It has been shown that exercise improves sleep quality as well as anti-depressive response in patients with chronic insomnia (Passos et al., 2014). It is not unreasonable to assume that subclinical disorders of mood and/or sleep are in part responsible for our findings.

Although a relationship between low EEG power and neurodegeneration has been established (Petit et al., 2004) it remains difficult to point to an exact biological mechanism that explains the benefits of exercise on sleep. It is known that areas of the brain associated with learning and memory (i.e., hippocampus) can increase in size with 12 weeks of exercise in younger individuals (Nauer et al., 2020) this was also associated with an enhanced performance on memory tasks. It is not unreasonable to think that these changes could also happen in elderly subjects. In fact, 16 weeks of aerobic exercise has been shown to prevent a decrease in hippocampal subfield volume in elderly subjects (Frodl et al., 2020). The potential for exercise to slow neurodegeneration through enhanced activity and prevention of synaptic and neuronal loss is an exciting avenue to investigate to optimize cognitive health. Several putative molecular mechanisms are described in a recent review on the subject (Santiago et al., 2022).

Aging-related decreases in cortical inhibition might be modified by 12-weeks of physical activity. Improvements in restorative sleep may aid in titrating anesthetic regimens in older participants. The loss of overall frontal EEG power has been shown to be an important predictor of adverse neurocognitive outcomes after surgery (Hesse et al., 2019). Therefore, it is conceivable that pre-habilitation with exercise could be a viable option for at-risk patients scheduled for surgery. Home-health devices as the one used in this study are a practical way to evaluate sleep quality in older adults, and in the future may be part of a comprehensive evaluation of cognitive health.

## Limitations

We are aware of our limited sample size and hence, further studies are needed to explore and interpret the changes in sleep macro-architecture and EEG patterns that are associated with an aerobic intervention. It remains possible that exercise can results in substantial improvement in perceptions of sleep quality in this population, however, we did not examine any subjective self-evaluations of sleep quality in our participants. Some researchers may have concerns about the auto-scoring software for classifying vigilance states. These devices show at least 80% agreement with expert raters and this is roughly equivalent to the amount of variability between individual raters (Levendowski et al., 2017).

## Data availability statement

The data analyzed in this study is subject to the following licenses/restrictions: The raw data supporting the conclusions of this article will be made available by the authors, without undue reservation. Requests to access these datasets should be directed to PG.

## Ethics statement

The studies involving human participants were reviewed and approved by the Institutional Review Board, Emory School of Medicine. The patients/participants provided their written informed consent to participate in this study.

## Author contributions

TC: data quality, coordination, interpretation, analysis, and draft and revision of the manuscript. KM and JN: overall project design, data collection, and draft and revision of the manuscript. VG, CS, and MK: data analysis, and draft and revision of the manuscript. PG: overall project design, data collection, interpretation, analysis, and draft and revision the manuscript. All authors contributed to the article and approved the submitted version.

## Footnotes

1
https://cportal.b-alert.com/sleep-profiler/documentshelp

## Supplementary material

Supplementary Figure 1 Exemplary hypnogram of the classical vigilance states **(A)** and density spectral array **(B)** of the pre-intervention EEG recording session.

Supplementary Figure 2 Power spectral density (PSD) plots for deep sleep, both pre- (blue) and post- (orange) aerobic intervention (*n* = 5). A histogram for the measured EEG power in each frequency bin is displayed. Solid lines represent average for all five participants, and shading represents 95% confidence interval. In deep sleep, we see an increase in EEG power post-exercise, especially at moderate to high frequencies. In addition, the AUC values and 95% bootstrapped CIs are presented.

Supplementary Figure 3 For light sleep and deep sleep. During deep sleep, the power in the low (delta) frequencies was significantly higher and the power in the high (beta) frequencies was significantly lower. In addition, the AUC values and 95% bootstrapped CIs are presented.

Supplementary Table 1 Statistical results corresponding to the figures.
