# Supplementary figures and images for: Effects of exercise on the sleep microarchitecture in the aging brain: A study on a sedentary sample
Source: Front Syst Neurosci. 2022 Oct 26;16:855107. doi: 10.3389/fnsys.2022.855107 (PMC9644157; doi:10.3389/fnsys.2022.855107)

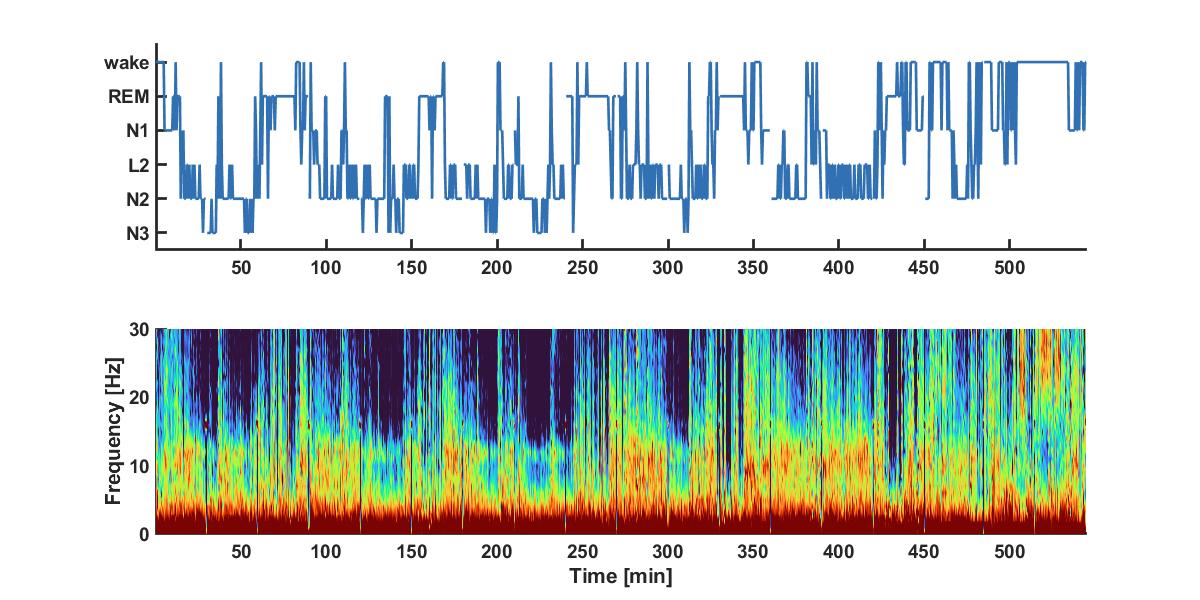

Supplement: Supplementary Figure 1 — Exemplary hypnogram of the classical vigilance states (A) and density spectral array (B) of the pre-intervention EEG recording session. [file Image_1.JPEG]

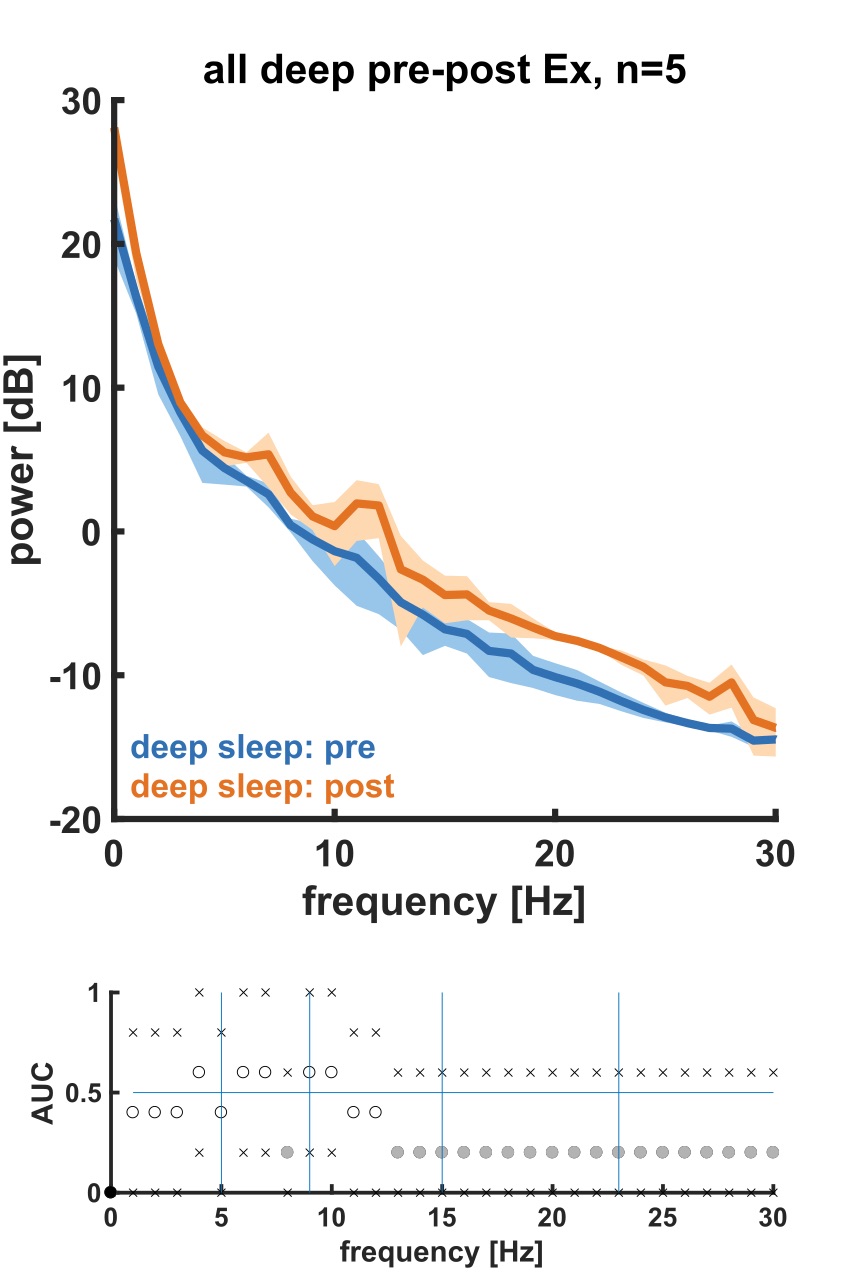

Supplement: Supplementary Figure 2 — Power spectral density (PSD) plots for deep sleep, both pre- (blue) and post- (orange) aerobic intervention (n = 5). A histogram for the measured EEG power in each frequency bin is displayed. Solid lines represent average for all five participants, and shading represents 95% confidence interval. In deep sleep, we see an increase in EEG power post-exercise, especially at moderate to high frequencies. In addition, the AUC values and 95% bootstrapped CIs are presented. [file Image_2.JPEG]

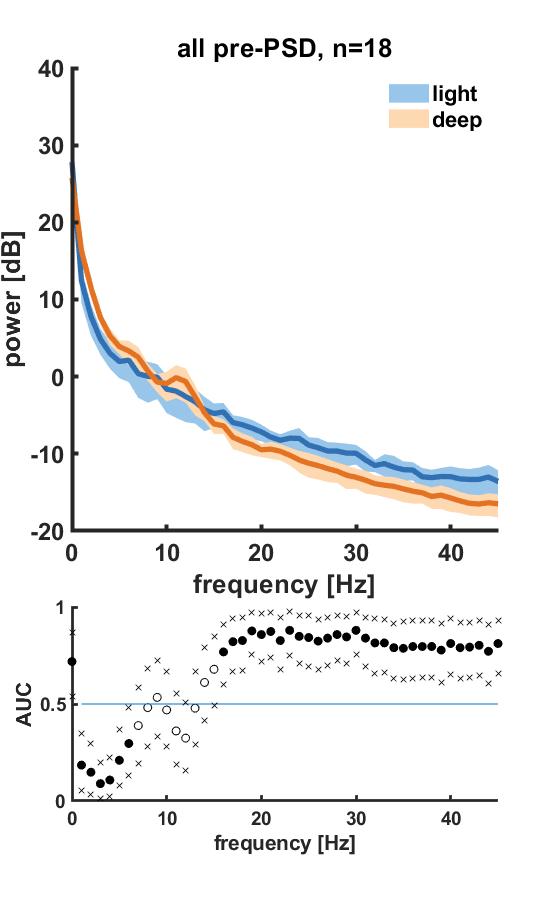

Supplement: Supplementary Figure 3 — For light sleep and deep sleep. During deep sleep, the power in the low (delta) frequencies was significantly higher and the power in the high (beta) frequencies was significantly lower. In addition, the AUC values and 95% bootstrapped CIs are presented. [file Image_3.JPEG]
