# Supplementary material for: Effects of exercise on the sleep microarchitecture in the aging brain: A study on a sedentary sample
Source: Front Syst Neurosci. 2022 Oct 26;16:855107. doi: 10.3389/fnsys.2022.855107 (PMC9644157; doi:10.3389/fnsys.2022.855107)
Supplement: Supplementary Table 1 — Statistical results corresponding to the figures. [file Table_1.DOCX]

| Figure 2B: recording time | |  |  | Figure 2A |  |  |  |
| --- | --- | --- | --- | --- | --- | --- | --- |
|  |  |  |  | **Friedman** |  |  | p<0.001 |
| Friedman: p<0.001 | |  |  | post hoc | wake | N1 | 0.9978 |
| Multcomp |  |  |  |  | wake | N2 | 0.125 |
| light | deep | 0.077 |  |  | wake | N3 | 0.1007 |
| light | REM | 0.2181 |  |  | wake | L2 | 0.9372 |
| deep | **REM** | **0.0004** |  |  | wake | REM | 1 |
|  |  |  |  |  | **N1** | **N2** | **0.0383** |
|  |  |  |  |  | N1 | N3 | 0.2667 |
|  |  |  |  |  | N1 | L2 | 0.9965 |
| Figure A: recording time | |  |  |  | N1 | REM | 0.9996 |
|  |  |  |  |  | **N2** | **N3** | **0** |
| Friedman: p<0.001 | |  |  |  | **N2** | **L2** | **0.0079** |
| wake | N1 | 0.9978 |  |  | N2 | REM | 0.0901 |
| wake | N2 | 0.125 |  |  | N3 | L2 | 0.5655 |
| wake | N3 | 0.1007 |  |  | N3 | REM | 0.1388 |
| wake | L2 | 0.9372 |  |  | L2 | REM | 0.9672 |
| wake | REM | 1 |  |  |  |  |  |
| N1 | **N2** | **0.0383** |  | Figure 2B |  |  |  |
| N1 | N3 | 0.2667 |  | **Friedman** |  |  | p<0.001 |
| N1 | L2 | 0.9965 |  | post hoc | light | deep | 0.077 |
| N1 | REM | 0.9996 |  |  | light | REM | 0.2181 |
| N2 | **N3** | **0** |  |  | **deep** | **REM** | **0.0004** |
| N2 | **L2** | **0.0079** |  |  |  |  |  |
| N2 | REM | 0.0901 |  | Figure 2C |  |  |  |
| N3 | L2 | 0.5655 |  | fractions pre | |  |  |
| N3 | REM | 0.1388 |  | **Friedman** |  |  | p=0.243 |
| L2 | REM | 0.9672 |  | fractions post | |  |  |
|  |  |  |  | **Friedman** |  |  | p=0.338 |
|  |  |  |  |  |  |  |  |
|  |  |  |  | Figure 2D |  |  |  |
| Figure C |  |  |  | fractions pre | |  |  |
| fractions pre | |  |  | **Friedman** |  |  | p=0.091 |
| Friedman: p=0.243 | |  |  | fractions post | |  |  |
|  |  |  |  | **Friedman** |  |  | p=0.091 |
| fractions post | |  |  |  |  |  |  |
| Friedman: p=0.338 | |  |  |  |  |  |  |
|  |  |  |  |  |  |  |  |
|  |  |  |  |  |  |  |  |
| Figure D |  |  |  |  |  |  |  |
| fractions pre | |  |  |  |  |  |  |
| Friedman: p=0.091 | |  |  |  |  |  |  |
|  |  |  |  |  |  |  |  |
| fractions post | |  |  |  |  |  |  |
| Friedman: p=0.091 | |  |  |  |  |  |  |
